# Generalized Vision-Based Detection, Identification and Pose Estimation of Lamps for BIM Integration

**DOI:** 10.3390/s18072364

**Published:** 2018-07-20

**Authors:** Francisco Troncoso-Pastoriza, Javier López-Gómez, Lara Febrero-Garrido

**Affiliations:** 1 School of Industrial Engineering, University of Vigo, Campus Universitario, 36310 Vigo, Spain; javilopez@uvigo.es; 2 Defense University Center, Spanish Naval Academy, Plaza de España, s/n, 36920 Marín, Spain; lfebrero@cud.uvigo.es

**Keywords:** building lighting, lamp detection, pose estimation, building information modelling

## Abstract

This paper introduces a comprehensive approach based on computer vision for the automatic detection, identification and pose estimation of lamps in a building using the image and location data from low-cost sensors, allowing the incorporation into the building information modelling (BIM). The procedure is based on our previous work, but the algorithms are substantially improved by generalizing the detection to any light surface type, including polygonal and circular shapes, and refining the BIM integration. We validate the complete methodology with a case study at the Mining and Energy Engineering School and achieve reliable results, increasing the successful real-time processing detections while using low computational resources, leading to an accurate, cost-effective and advanced method. The suitability and the adequacy of the method are proved and concluded.

## 1. Introduction

Lighting accounts for approximately 19% of the electricity consumed all over the world [1], but there are great possibilities of achieving savings by replacing inefficient lighting sources [2,3]. Indeed, over the past decade, the worldwide demand for artificial lighting increased at an average rate of 2.4% per year [1]. In buildings, artificial lighting is a significant contributor to energy consumption and costs, consuming the highest electrical energy, approximately one-third of the electricity used [3,4,5]. Therefore, the knowledge of the real lighting inventory and conditions and the adequate management of lighting systems are crucial when addressing energy conservation measures (ECMs) [5]. Not only does this knowledge allow us to reduce energy consumption, but it can also save money for the building’s owners [3]. Consequently, the building lighting must be accurately known and then reliably integrated into the building information modelling (BIM).

BIM is a technology widely recognized and increasingly investigated in the architecture, engineering and construction (AEC) industry [6,7,8]. BIM can be defined as “a set of interacting policies, processes and technologies producing a methodology to manage essential building design and project data in digital format throughout the building’s lifecycle” [9]. It represents the digital model of the building as an integrated and coordinated database that enables sharing and transferring information about the whole building [8]. BIM tools are designed mainly for the analysis of multiple performance criteria, including lighting as a main issue [7,8,10]. Typically, BIM software implements internally a lighting condition analysis, differentiating between the natural and artificial lighting [7]. However, the main obstacle is the lack of accurate information [7]. The work presented in this article tries to solve this issue by looking for new methods that allow the accurate identification and state of lamps. Although research on the building lighting related to BIM has been deeply addressed by many authors [8,11,12,13], the integration of computer vision is relatively new [14].

Computer vision is a technology of obtaining and evaluating a digital image to acquire a certain type of information and can be widely applied [15]. Moreover, computer vision is helpful to shorten the time-consuming inspection process [15,16]. Computer vision systems (CVSs) have progressed and currently focus on depth data besides edge-based image algorithms. Nevertheless, edge-based image algorithms still lead to better outcomes for object detection and location in many cases [17]. Methods for object detection, location and 3D pose estimation have been comprehensively explained in a previous article [14], classifying them into image-based [18] and model-based techniques [19] suitable for textureless object detection. Matching is a key problem in the digital image analysis, and edges are perhaps the most important low-level image feature [20]. Chamfer matching algorithms are high-performance solutions to the shape-based object detection, which calculate distances between edges, and the chamfer distance transform has been effectively used in model-based methods for the edge-based matching [14]. State-of-the-art and different chamfer distance transform algorithms are gathered and explained in depth [14]. The new procedure proposed in this work outperforms other methods in different aspects. The new procedure is an improved version of our previous work, which enhances the candidate and model selection while leveraging the fast directional chamfer matching (FDCM) [21] and the pose refinement and scoring of the direct directional chamfer optimization (D${}^{2}$CO) [17].

Image registration is a process of overlapping two or more images of the same scene taken at different times, from different perspectives, and by different sensors [22]. Typically, image registration is required in remote sensing, medicine, cartography and computer vision [22]. Several authors have applied this to detect lighting and lamps. Elvidge et al. [23] investigated the optimal spectral bands for the identification of lighting types and estimated four major indices to measure the efficiency of lighting, which lead to good results with minimal spectral overlap. Liu et al. [24] proposed an imaging sensor-based light emitting diode (LED) lighting system that implemented a finer perception of the environmental lighting, resulting in a more precise lighting control. Ng et al. [15] presented an integrated approach combining a CVS and real-time management system (RTMS) to solve quality control problems in the manufacturing of lighting products.

This work proposes a complete and novel methodology based on computer vision to detect, identify and locate all types of lamps independently of the shape of their light surface. We describe the design and the development of new algorithms that enhance current methods in the literature using computer vision and imaging processing tools. The results from the whole system, which is suitable for any type of lamp shape, are integrated into a BIM with the aim of solving problems related to time-consuming operations and human errors. The main contribution of this work lies in the generalization of the shape and pose estimation techniques to allow the identification of a much wider range of lamp shapes and the improvements in the localization system and the BIM integration step. This work applies a novel technology in a fast and practical way, therefore innovating the building lighting. However, the applications can be extended to other sectors given the cross-sectional nature of the method. In addition, the method can be widely used in the continuous and automatic scanning of lamps, the precise knowledge of the state of a lamp, the establishment of a lamp stock, the electrical facility maintenance, the energy audit and the setting of conformable indoor conditions for the occupants.

## 2. Materials and Methods

**Figure 1:**
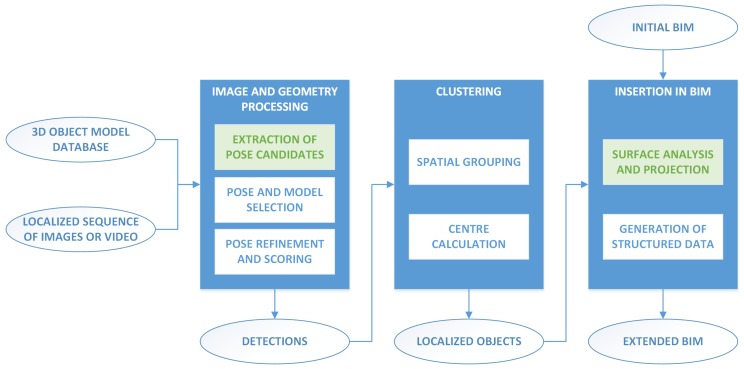
General diagram of the complete BIM generation process.

The methodology proposed in this work is based on three main steps: image and geometry processing, clustering, and insertion in the BIM. Figure 1 shows a general diagram of this whole process. In the first step, the input images are analysed to obtain initial pose candidates based on the detected shapes. Then, for each detection, a lamp model is selected leveraging the FDCM [21] based on the available edge information of the image extracted using the line segment detector (LSD) [25]. Lastly, the pose is refined using the D${}^{2}$CO [17]. In the second step, a clustering operation is performed on the set of individual detections, and a centre is calculated for each of the resulting clusters, leading to a collection of localized objects. In the last step, the information from the detected objects is inserted into the BIM model of the building, assigning the detections to the corresponding space.

We introduce the following major enhancements to our previous work [14]: (i) the generalization of the shape and pose estimation to automatically detect polygonal shapes with different numbers of sides and elliptical shapes; and (ii) the use of the available BIM information in the final insertion step by means of a surface projection method. These improvements yield more refined results and provide a wider range of application.

The complete system and each of the custom algorithms presented in this work have been developed in C++, with the help of the following supporting software libraries: OpenCV [26] for general artificial vision algorithms, OpenMesh [27] to read and process the 3D geometric information of object models, Ceres Solver [28] to solve the different optimization problems involved in the method, and OpenGL [29] to obtain the occlusion information on the 3D projections.

### 2.1. Generalized Shape and Pose Estimation

In our previous work [14], we introduced an algorithm to obtain the shape and the pose of objects projecting a quadrilateral on the image. Here, we generalize the shape estimation to automatically detect the number of sides of the final polygon, with the possibility of also detecting elliptical shapes, and introduce the necessary changes to the pose estimation to be compatible with either polygonal or elliptical shapes. We use the term *pose* to denote a rigid transformation of an object, composed of a vector in $R^{3}$ that determines the translation and a vector in so(3)—the Lie algebra associated with the special orthogonal group SO(3)—that determines the orientation.

#### 2.1.1. Polygon Estimation

The method presented in [14] aims to obtain an estimation of the shape of a polygon with a fixed number of sides *k* based on an initial contour with n>k sides. The method is an extension of the work of Visvalingam et al. [30] for strictly inner, strictly outer, or general polygons, based on a predefined score function. Here, we use an area-based score function to detect polygons with an arbitrary number of sides based on a threshold $a_{\max}$ as the termination criterion. This method is presented in Algorithm 1 with the additional functions in Algorithm 2. We use the method of Sklansky [31] to make the initial contour convex. The method stops when the next best area relative to the original contour area is greater than $a_{\max}$. This method is based on the fact that the reduction of the area should be relatively small until the final number of sides is reached, at which point there should be a noticeable increase in the area reduction.

**Algorithm 1** Fit polygon.
**Require:**
   $P=\{p_{k}\}$ is a sequence of *n* points   $a_{\max}$ is the area threshold to stop removing sides
**Ensure:**
   $F=\{f_{k}\}$ is a sequence of *m* points representing an approximated polygon for *P*
  1:**function**FitPolygon(P, $a_{\max}$)  2:    F←ConvexHull(*P*)                        ▹ From [31]  3:    A←Area(F)  4:    $R=\left\{r_{k}\right\}\leftarrow {\left\{0,\ldots ,0\right\}}_{n}$; $S=\left\{s_{k}\right\}\leftarrow {\left\{0,\ldots ,0\right\}}_{n}$; $Q=\left\{q_{k}\right\}\leftarrow {\left\{0,\ldots ,0\right\}}_{n}$  5:    **for** k←1,lenF **do**  6:          $r_{k}\leftarrow$InnerScore(F, *k*)                      ▹ Algorithm 2  7:          $s_{k},q_{k}\leftarrow$OuterScore(F, *k*)                    ▹ Algorithm 2  8:    **end for**  9:    **while** true **do**10:          $i\leftarrow \arg\;\min\{r_{k}\}$; $j\leftarrow \arg\;\min\{s_{k}\}$11:          $a\leftarrow \min(r_{i},s_{j})$12:          **if** $a/A>a_{\max}$ **then**13:               **break**14:          **end if**15:          $l\leftarrow \arg\;\min(r_{i},s_{j})$16:          RemoveElement(*R*, *l*); RemoveElement(*S*, *l*); RemoveElement(F, *l*)17:          **if** $s_{i}<r_{j}$ **then**18:               $f_{l}\leftarrow q_{l}$19:          **end if**20:          RemoveElement(Q, *l*)21:          $r_{l-1}\leftarrow$InnerScore(F, l−1); $r_{l}\leftarrow$InnerScore(F, *l*)         ▹ Algorithm 222:          $s_{l-1},q_{l-1}\leftarrow$OuterScore(F, l−1); $s_{l},q_{l}\leftarrow$OuterScore(F, *l*)    ▹ Algorithm 223:          **if** $s_{i}<r_{j}$ **then**24:               $r_{l+1}\leftarrow$InnerScore(F, l+1)                 ▹ Algorithm 225:          **else**26:               $s_{l-2},q_{l-2}\leftarrow$OuterScore(F, l−2)              ▹ Algorithm 227:          **end if**28:    **end while**29:    **return** F30:
**end function**



**Algorithm 2** Score functions.
  1:**function**InnerScore(F, *k*)  2:      **return** Area($f_{k-1}$, $f_{k}$, $f_{k+1}$)  3:
**end function**
  4:   5:**function**OuterScore(F, *k*)  6:      **if** $\left(f_{k}-f_{k-1}\right)\times \left(f_{k+1}-f_{k+2}\right)>0$ **then**  7:          p←Intersection($f_{k-1}$, $f_{k}$, $f_{k+1}$, $f_{k+2}$)  8:          a←Area($f_{k}$, p, $f_{k+1}$)  9:      **else**10:          p←0; a←∞11:      **end if**12:      **return** *a*, p13:
**end function**



#### 2.1.2. Shape Estimation

The polygon estimation method is included in a more general shape and pose estimation technique presented in Algorithm 3. First, we obtain a coefficient to determine if the shape is polygonal or elliptical based on a predefined threshold sth. In the first case, we estimate the polygon using Algorithm 1; in the second case, we use the method introduced by Fitzgibbon and Fisher [32] to obtain the final shape parameters.

**Algorithm 3** Fit shape.
**Require:**
   $P=\{p_{k}\}$ is a sequence of *n* points   $a_{\max}$ is the area threshold to stop removing sides   sth is the maximum number of sides for the shape to be considered a polygon   $M=\{m_{i}\}$ is a set of object models   C are the parameters of the camera model
**Ensure:**
   P={Π} is a set of estimated poses for objects in M based on the contour information
  1:**function**FitShape(P, $a_{\max}$)  2:      **if** ShapeCoefficient(F)>sth **then**                ▹ Section 2.1.2  3:          F←FitPolygon(P)                      ▹ Algorithm 1  4:          **for all** $m_{i}\in M$ **do**  5:                **if** $m_{i}$ has a non-circular shape **then**  6:                    $\Pi_{i}\leftarrow$SolvePnp(F, *C*, $m_{i}$)  7:                **end if**  8:          **end for**  9:      **else**10:          $F=\{f_{\mathrm{up}},f_{\mathrm{right}},f_{\mathrm{down}},f_{\mathrm{left}}\}\leftarrow$FitEllipse(F)          ▹ From [32]11:          **for all** $m_{i}\in M$ **do**12:                **if** $m_{i}$ has a circular shape **then**13:                    $\Pi_{i}\leftarrow$EstimateCircular(F, P, *C*, $m_{i}$)           ▹ Section 2.1.314:                **end if**15:          **end for**16:      **end if**17:      **return** F, C18:
**end function**



The shape coefficient *s* is obtained based on the circularity [33] of the shape as follows:

(1) $$\begin{array}{c} s=\frac{p^{2}}{a}, \end{array}$$

being *p* the shape perimeter and *a* its area.

The aim is to obtain higher values for polygons compared to those for ellipses.

#### 2.1.3. Pose Estimation

We use two different methods to estimate the pose based on the image shape. In the case of a polygon, we solve a PnP (*Perspective-n-Point*) problem using an iterative method based on the Levenberg–Marquardt optimization [34,35] as described in [14]. However, if the shape is elliptical, we do not have a direct correspondence between points in 2D and in 3D. We could use the four axis points from the projected ellipse, but Luhmann [36] showed that the eccentricity in the projection of circular target centres should not be ignored in real applications. Therefore, we have to modify the classic PnP problem to account for the absence of a direct correspondence. Using the contour points from the image, we formulate a minimization problem based on the distance of the projected image points on the circle plane to its circumference.

Let E be an ellipse with a centre $q_{E}=\left(u_{E},v_{E}\right)$, a semi-major axis of length *a* and a semi-minor axis of length *b*, rotated by an angle θ. Let C be a circle for which *E* is a projection on the image plane, with a centre $p_{C}=\left(x_{C},y_{C},z_{C}\right)$ and a radius $R_{C}$, included in the plane P with a unit normal vector $\hat{n}=\left(x_{n},y_{n},z_{n}\right)$. Let K be the matrix of the intrinsic parameters of the camera:

(2) $$\begin{array}{c} K=\left[\begin{array}{ccc} f_{x} & 0 & c_{x}\\ 0 & f_{y} & c_{y}\\ 0 & 0 & 1 \end{array}\right], \end{array}$$

with focal lengths $f_{x}$ and $f_{y}$, and principal point offsets $c_{x}$ and $c_{y}$.

For each point $q_{i}=\left(u,v\right)$ on the contour of the ellipse, we can obtain its corresponding position $p_{i}=\left(x_{i},y_{i},z_{i}\right)$ in the camera coordinate system on the plane z=1 as

(3) $$\begin{array}{c} p_{i}=K^{-1}\left[\begin{array}{c} q_{i}\\ 1 \end{array}\right]=\left[\begin{array}{ccc} 1/f_{x} & 0 & -c_{x}/f_{x}\\ 0 & 1/f_{y} & -c_{y}/f_{y}\\ 0 & 0 & 1 \end{array}\right]\left[\begin{array}{c} u\\ v\\ 1 \end{array}\right]=\left[\begin{array}{c} \left(u-c_{x}\right)/f_{x}\\ \left(v-c_{y}\right)/f_{y}\\ 1 \end{array}\right], \end{array}$$

Let L be the projection line from the camera origin to the point $p_{i}$. The intersection point $p_{i}^{\prime }$ between this line and the circle plane is given by their corresponding equations:

(4) $$\begin{array}{c} \left\{\begin{array}{c} L:p_{i}^{\prime }=zp_{i}\\ P:\hat{n}\left(p_{i}^{\prime }-p_{C}\right)=0 \end{array}\right.\to \;p_{i}^{\prime }=p_{i}\frac{\hat{n}p_{C}}{\hat{n}p_{i}} \end{array}$$

Then, for each point, we try to minimize the distance from its projection to the circumference:

(5) $$\min_{p_{C},n}\;\sum_{i}{\left(∥p_{i}\frac{\hat{n}p_{C}}{\hat{n}p_{i}}-p_{C}∥-R_{C}\right)}^{2}$$

(6) $$\begin{array}{cc} s.t. & \;∥\hat{n}∥=1 \end{array}$$

As for the classic PnP problem, we solve the minimization using an iterative method based on the Levenberg–Marquardt optimization [34,35]. The constraint on the unit normal vector is taken into account by performing a local parameterization of $\hat{n}$ in the tangent space of the unit sphere.

To improve the convergence of the method, we adopt the following initial guess of $p_{C}$ and $\hat{n}$:

(7) $$\begin{array}{cc} p_{C}^{\left(0\right)}= & \frac{R_{C}}{\sqrt{ab}}p_{E} \end{array}$$

(8) $$\begin{array}{cc} {\hat{n}}^{\left(0\right)}= & \left[\begin{array}{c} m_{x}\sqrt{1-{\left(b/a\right)}^{2}}\\ m_{y}\sqrt{1-{\left(b/a\right)}^{2}}\\ b/a \end{array}\right] \end{array}$$

being $p_{E}$ the corresponding position of $q_{E}$ in the camera coordinate system on the plane z=1 and $\hat{m}=\left(m_{x},m_{y},0\right)$ a unit vector along the direction of the minor axis of the projected ellipse.

Lastly, we obtain the rotation vector from the resulting unit normal vector of the plane as follows:

(9) $$\begin{array}{cc} r^{\prime } & {=[0,0,1]}^{t}\times \hat{n}, \end{array}$$

(10) $$\begin{array}{cc} r & =r^{\prime }\frac{\arcsin∥r^{\prime }∥}{∥r^{\prime }∥}. \end{array}$$

### 2.2. Surface Projection in the BIM Integration

The BIM model of the building represents an additional source of information that can be used to improve the accuracy of the detections. Apart from the insertion of the new data exemplified in [14], we can also use the geometric information from the BIM model to extract a list of surfaces with spatial information and use them to adjust the positions of the detections. Assuming gbXML [37]—an open schema created to facilitate the transference of building data stored in BIM to engineering analysis tools—as the supporting format for the BIM information, we can obtain the required data by accessing the elements with path “gbXML/Campus/Surface/PlanarGeometry” in the XML tree.

Given that the detected lamps are embedded in the ceilings of the building, we can perform a projection in the 3D space of each of the detections to the nearest building surface. Let $S=\{s_{i}\}$ be the set of the surfaces of a building model, each one with a unit normal vector ${\hat{n}}_{k}$ and a point $x_{k}$ included in the plane defined by the surface. Then, the surface in the model that is the closest to a point p is given by

(11) $$\begin{array}{c} K=\underset{k}{\arg\min}\;|d_{k}|=\underset{k}{\arg\min}\;\left|{\hat{n}}_{k}\cdot \left(p-x_{k}\right)\right|. \end{array}$$

Then, the projected location $p^{\prime }$ of a detection positioned at p, with the nearest model surface $s_{K}$ at a distance $d_{K}$ and with a unit normal vector ${\hat{n}}_{K}$, is

(12) $$\begin{array}{c} p^{\prime }=p-d_{K}{\hat{n}}_{K}. \end{array}$$

With this method, we can improve the location of the detections and, at the same time, assign the detections to the corresponding space in the building model based on the nearest surface. This is a more effective and simpler approach compared to the point-in-polyhedron test used in [14].

## 3. Description of the Experimental System

**Figure 2:**
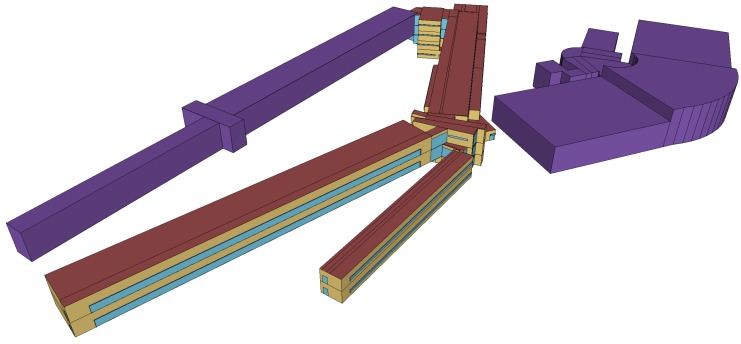
Geometry of the BIM model of the Mining and Energy Engineering School of the University of Vigo in SketchUp.

**Figure 3:**
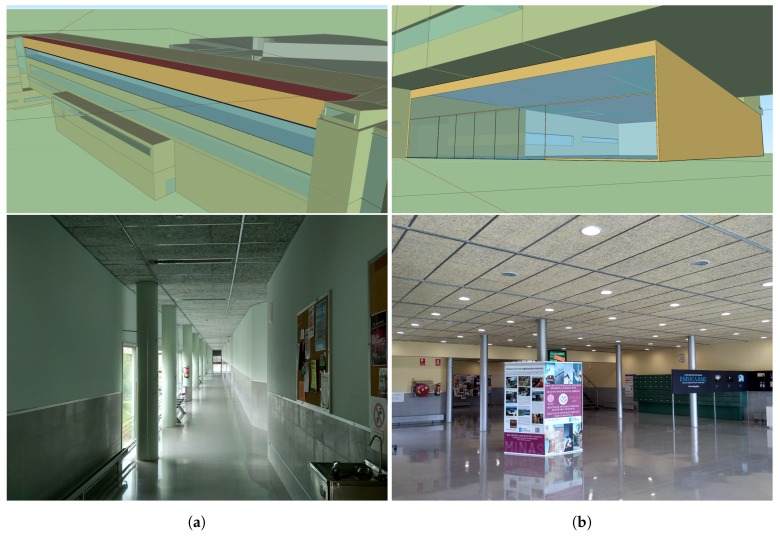
Spaces used in the experiments: (**a**) classroom corridor, rectangular lamps (Model 4); and (**b**) hall, circular lamps (Model 5).

The acquisition of the experimental data took place in two locations at the Mining and Energy Engineering School of the University of Vigo in Spain. Figure 2 shows the geometry of the BIM model of this building. The two locations used for our tests are displayed in Figure 3. The first one consists of a corridor of a classroom area with rectangular lamps, while the second one is a hall with circular lamps. Both lamp types are embedded in the ceiling.

**Figure 4:**
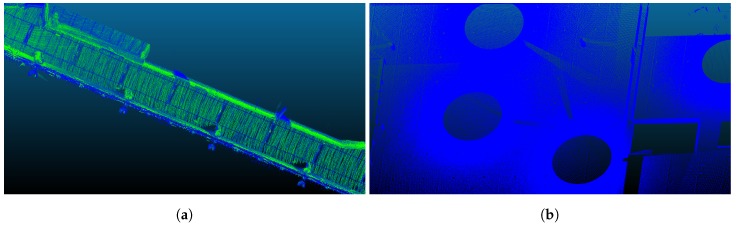
Point clouds for the two spaces used as ground truth for the experimental results: (**a**) classroom corridor, rectangular lamps (Model 4), extracted with the backpack system; and (**b**) hall, circular lamps (Model 5), extracted with FARO Focus3D X 330.

We used point clouds extracted with high-accuracy sensors as the ground truth for our experiments for the position of the lamps. These clouds are shown in Figure 4. The cloud in Figure 4a was obtained using a backpack-based inspection system based on LiDAR sensors and inertial measurement unit (IMU), whose data were processed with simultaneous localization and mapping (SLAM) techniques [38,39]. The second cloud, in Figure 4b, was captured with a FARO Focus3D X 330 Laser Scanner from FARO Technologies Inc. (Lake Mary, FL, USA). The technical characteristics of both systems are presented in Table 1.

**Figure 5:**
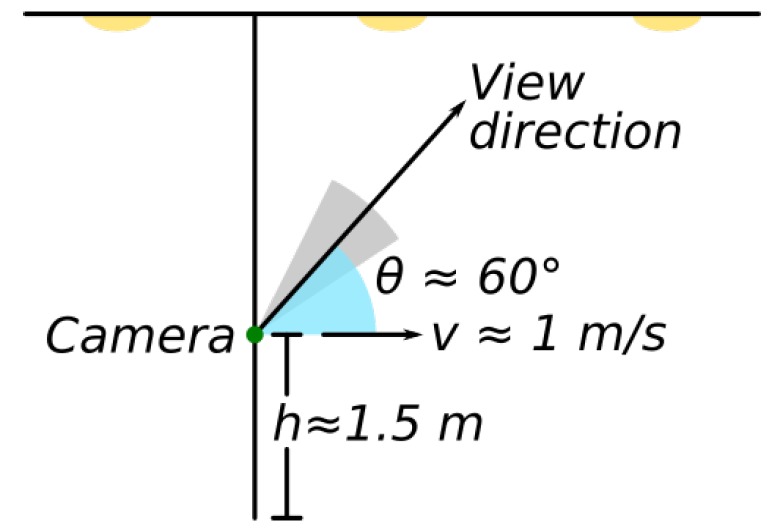
Measurement parameters used in the experiments. The acquisition process with the Lenovo Phab 2 Pro was done at a normal walking speed of around 1 m/s, at 1.5 m from the floor with a pitch of approximately 60${}^{{}^{\circ}}$.

We obtained the greyscale images and the location data for the two places using a Lenovo Phab 2 Pro with Google Tango [40]. The images were extracted at an approximate rate of 30 frames per second and had an original resolution of 1920 × 1080 but were later downscaled to 960 × 540 before the processing to improve the speed of the method. The location data were obtained from the information provided by the IMU of the device combined with the visual features of the environment using advanced computer vision and image processing techniques to improve the accuracy of the motion tracking information [40]. Some statistics of the complete dataset of images and the two locations used in the experiments are displayed in Table 2. The acquisition process, depicted in Figure 5, was done at a walking speed of ≈1 m/s, positioning the camera at 1.5 m from the floor with a pitch of ≈60${}^{{}^{\circ}}$ with respect to the horizontal plane.

**Figure 6:**
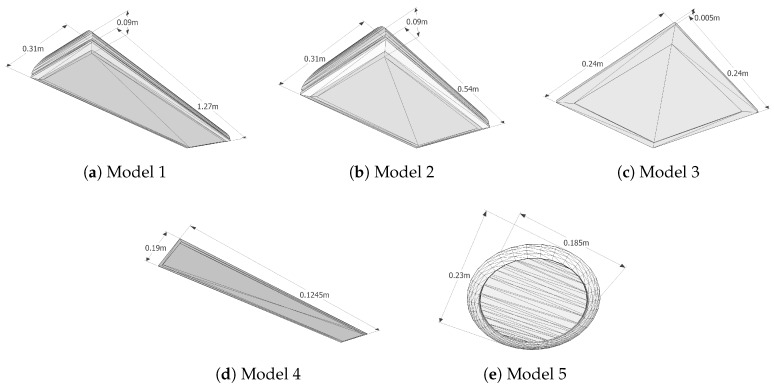
Lamp models in the database used in the experiments.

Regarding the 3D models, we added two new items to the ones presented in [14], corresponding to the lamps found in the locations of the experiments. With this addition, the geometric characteristics of all the elements in the database used for the experiments are shown in Figure 6, including the two new lamp models (Models 4 and 5). We keep the original three lamps to assess the identification capability of our system with additional models of similar geometries. The specifications of the lamp bulbs for each model are shown in Table 3.

## 4. Results and Discussion

**Figure 7:**
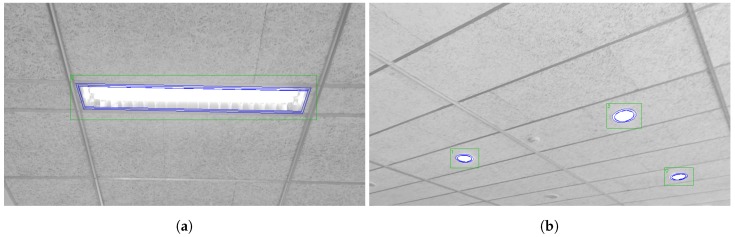
Examples of detections for different shapes: (**a**) rectangular; and (**b**) circular.

We performed tests for each of the technical contributions presented in this work. In this section, we show their outcomes as well as the final values for the new case study described in Section 3. Figure 7 includes some examples of the detections for this new case study for each lamp type.

### 4.1. Generalized Shape and Pose Estimation

**Figure 8:**
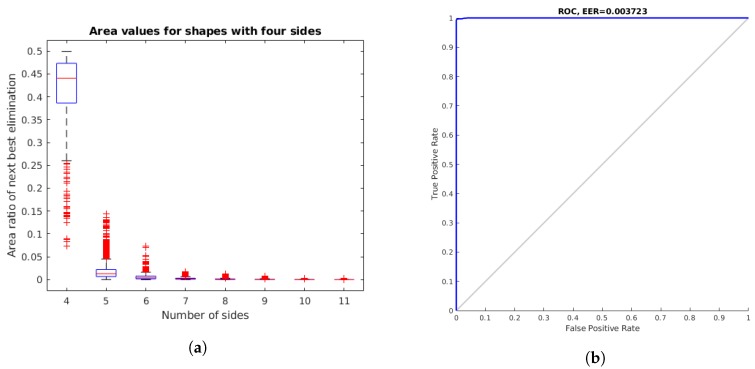
Area ratio of next best elimination for different sides of contours corresponding to shapes with four sides. Most of the polygonal shapes were correctly identified as quadrilaterals, with an equal error rate (EER) of 0.003723. (**a**) Boxplot; and (**b**) receiver operating characteristics for the classification of four-side shapes.

We verified the generalized polygon estimation technique presented in Section 2.1.1. Figure 8a shows the area ratio used to stop eliminating points in Algorithm 1 with respect to the number of sides for the shapes obtained from light surfaces with four sides. The light surface instances used in this test were obtained from the image test dataset, comprising a total of 1343 contours of rectangular lamps. As presented in Figure 8b, the great majority of the shapes were correctly classified as quadrilaterals, with an equal error rate (EER) of 0.003723. The few remaining shapes corresponded to very distorted light surface detections with a higher number of apparent sides.

**Figure 9:**
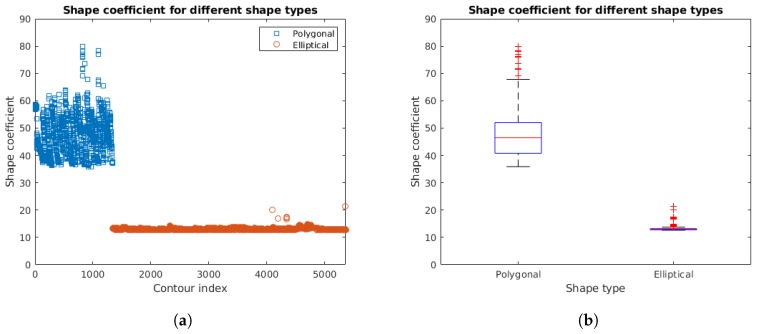
Shape coefficient presented in Equation (1) for different shape types: (**a**) results for individual contours; and (**b**) statistics of the combined results.

The second verification corresponds to the generalized shape estimation. The results of the shape coefficient of Equation (1) for the subset of light surface shapes in the dataset are shown in Figure 9b. This subset contains 1343 shapes corresponding to the rectangular lamps and 4020 corresponding to the circular lamps. We can see that all shapes were correctly classified as polygonal or elliptical for this dataset when we selected a shape threshold of 21.437<sth<35.869.

### 4.2. Identification

These results are related to the identification of the specific lamp model among the ones registered in the database. As previously mentioned, there are a total of five lamp models in the database, resulting in five target and output classes in the classification problem. However, the input consist of instances of Models 4 and 5 only, while the others are kept to test the ability of the system to identify the correct lamp even in the presence of additional models, verifying the validity of the system in a more realistic case of a potentially larger database with additional elements not included in an specific area of the building.

**Figure 10:**
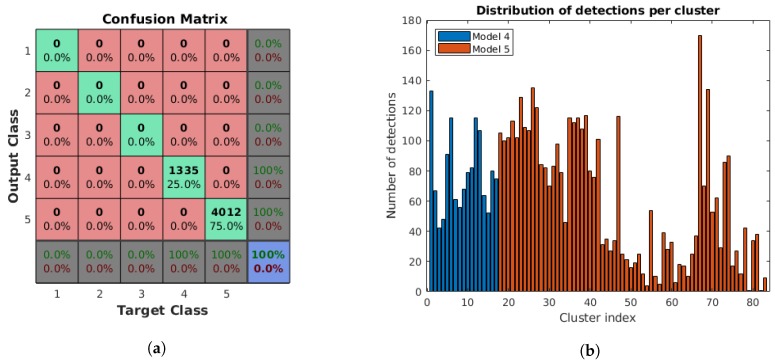
Results of the identification and clustering procedure. All detections were correctly classified, with neither incorrect detections of the three old models (Models 1, 2 and 3) nor errors between Models 4 and 5: (**a**) confusion matrix for the five lamp models used in the experiments; and (**b**) distribution of detections per cluster for each lamp model. Average number of elements: 78.53 for Model 4, 60.79 for Model 5, and 64.42 for all clusters.

Figure 10a shows the confusion matrix for the individual detections with the five classes corresponding to the five lamp models for a total of 1335 and 4012 detections of the rectangular (Model 4) and circular (Model 5) lamps, respectively. We can see that all detections were correctly classified, and, even when the three additional models were included, none of the detections were incorrectly identified as one of these, as shown in the first three rows/columns of the confusion matrix. Moreover, there are no errors between Model 4 and Model 5, which is expected from the results of the shape type classification procedure, with 100% correct classifications in the last two rows/columns of the confusion matrix.

Figure 10b illustrates the distribution of detections for each cluster. Some of the clusters for the circular lamps have a very low number of detections, due to the fast-moving blurred images or the low ambient lighting conditions that result in the target light being too bright, removing important edge information from the surrounding area. Nevertheless, the average number of detections per cluster is 64.42, which is sufficiently high to compensate the potential negative effect of outliers in the cluster.

### 4.3. Localization, State and Surface Projection

**Figure 11:**
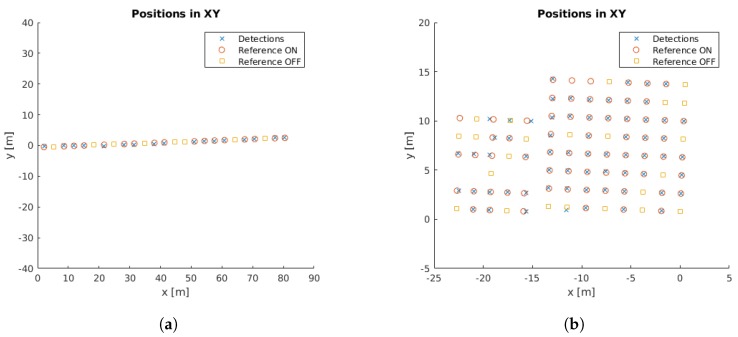
Position of detections and reference values with their corresponding ON/OFF state: (**a**) rectangular lamps; and (**b**) circular lamps.

These results are intended to quantify the errors in the localization outcome and the improvements of the surface projection method. Figure 11 shows the positions of all the cluster centres obtained from the detections of our system as well as the reference values based on the high-accuracy point clouds with their corresponding ON/OFF state. There should be one detection per turned on lamp; however, the lamps that are turned off should not be registered by the system to correctly identify the lamp state.

**Figure 12:**
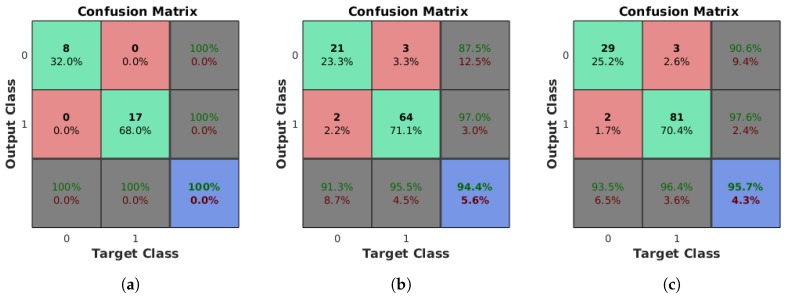
Confusion matrices for the ON/OFF state of the lamps for the different types. Class 0: turned off; Class 1: turned on. Overall, 100% of the rectangular lamps were correctly classified, while 5.6% of the circular lamps were assigned to the wrong class. The correct state was given for a total of 95.7% of the lamps of both models: (**a**) rectangular lamps; (**b**) circular lamps; and (**c**) all lamps.

Figure 12 presents the confusion matrices for the lamp state of the rectangular lamps, the circular lamps, and both, where Class 0 corresponds to the OFF state and Class 1 to the ON state. As shown in Figure 12a, the state of all rectangular lamps was captured accurately, while Figure 12b shows that there were some errors for the circular lamps: three of them were incorrectly detected as OFF, while two were incorrectly detected as ON. Altogether, 95.7% of the lamps were assigned to the correct state, as represented in Figure 12c.

**Figure 13:**
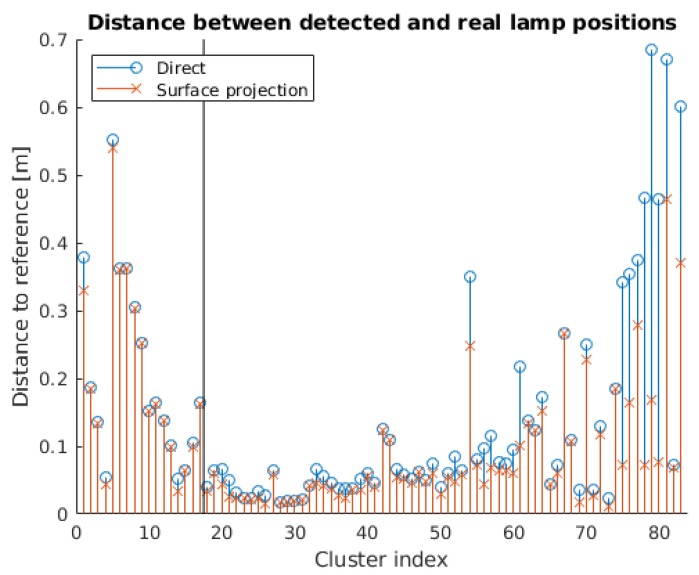
Distance between detected and real lamp positions with and without surface projection. The use of the surface projection step reduces the location error for all the detected lamps.

Regarding the localization of the lamps, Figure 13 shows the distance from the detected to the reference lamp positions. We include the results with and without the surface projection step. We can see that the use of the surface projection method reduces the distance to the reference values when assigning the detections to the corresponding BIM space. As displayed in Table 4, the error was reduced by 2.94% for the rectangular lamps, 36.0% for the circular lamps and 26.3% for the entire dataset.

## 5. Conclusions

We have presented a complete method for the automatic detection, identification and localization of the lamps to be directly integrated into the BIM of the building. The method is based on our previous work, extending its applicability to a much wider type of lamps and improving the integration method in the BIM. We have applied this method to a completely new case study with different lamp models to assess the performance benefits and the enhanced versatility accomplished with the introduction of the novel contributions.

The results show that there is a high percentage of polygonal shapes correctly identified as quadrilaterals, with an EER of 0.003723. Moreover, all 5363 light surface contours in the dataset are accurately classified as either polygonal or elliptical. Finally, the identification of 5347 detections has a 100% success rate, even when three additional models are kept in the database. With respect to the lamp state, there is a high percentage of correct classification, with 95.7% of the lamps assigned to the appropriate state. Additionally, the distance between the detected and actual lamp positions in the building is 14.54 cm on average and is reduced to 10.71 cm if the surface projection step is included, which results in a 26.3% decrease in the location error. Considering all the results obtained in the experiments, we have verified that the method can be applied to the intended use cases and that the new additions lead to better results in terms of the identification and the localization.

Our method relies only on single-image information; thus, a procedure to distinguish lamps with the same shape and different size does not exist. We are working on extensions to our methodology to overcome this limitation by leveraging the combined information of the same detection from different camera views and to also use the available depth information provided by the Tango platform. Moreover, if the BIM information is known beforehand, which can be used in prior steps of the methodology. Therefore, we are working on methods to utilize this information earlier to better adjust the data to the specific model for each of the individual detections and improve the overall accuracy of the results.

## Figures and Tables

**Table 1:** Technical characteristics of the two systems used to extract point clouds.

	Backpack System	FARO Focus3D X 330
**Max. range**	100 m	330 m
**Measurement rate**	300,000 points/s	122,000–976,000 points/s
**Step size (Vertical/Horizontal)**	2.0${}^{{}^{\circ}}$/0.1–0.4${}^{{}^{\circ}}$	0.009${}^{{}^{\circ}}$/0.009${}^{{}^{\circ}}$
**Field of View (Vertical/Horizontal)**	30${}^{{}^{\circ}}$/360${}^{{}^{\circ}}$	300${}^{{}^{\circ}}$/360${}^{{}^{\circ}}$
**Ranging error**	3 cm	2 mm

**Table 2:** Statistics of the dataset and the spaces used for the experiments.

	Rectangular	Circular	Total
**No. images**	6082	17,410	23,492
**No. light surface shapes**	1343	4020	5363
**No. image detections**	1335	4012	5347
**No. global detections (clusters)**	17	66	83
**No. lamps**	25	90	115
**No. lamps turned on**	17	67	84

**Table 3:** Characteristics of the bulbs in the lamp models.

Model	No.	Brand	Series	Tech.	Power	Brightness	Color
1	2	Osram	Lumilux	Fluor.	36 W	3350 lm	4000 K
2	2	Sylvania	Lynx	Fluor.	36 W	2800 lm	3000 K
3	2	Osram	Dulux	Fluor.	26 W	1800 lm	4000 K
4	1	Philips	TL-D	Fluor.	36 W	2500 lm	6200 K
5	2	Adolfo Alba	L01	Fluor.	26 W	2200 lm	4000 K

**Table 4:** Average distance between detections and reference values.

	Rectangular	Circular	All
**Direct**	20.75 cm	12.94 cm	14.54 cm
**Surface projection**	20.14 cm	8.28 cm	10.71 cm
**Error reduction**	**2.94%**	**36.0%**	**26.3%**

## Abbreviations

The following abbreviations are used in this manuscript:

ECM	Energy conservation measures
BIM	Building information modelling
AEC	Architecture, engineering and construction
CVS	Computer vision system
RTMS	Real-time management system
FDCM	Fast directional chamfer matching
D${}^{2}$CO	Direct directional chamfer optimization
LED	Light emitting diode
PnP	Perspective-n-Point
gbXML	Green Building XML Schema
IMU	Inertial measurement unit
SLAM	Simultaneous localization and mapping
ROC	Receiver operating characteristics
EER	Equal error rate
